# Hereditary Renal Cell Carcinoma: Is Age an Independent Criterion for Genetic Testing? A Large Cohort from a Latin America Referral Center

**DOI:** 10.15586/jkcvhl.v10i3.242

**Published:** 2023-08-01

**Authors:** Tomás Carminatti, Patricio Aitor García Marchiñena, Ignacio Pablo Tobia González, Valeria de Miguel, Marcelo Martín Serra, Pablo Germán Kalfayan, Alberto Manuel Jurado

**Affiliations:** 1 Urology Department, Hospital Italiano de Buenos Aires, Buenos Aires, Argentina;; 2 VHL unit, Hospital Italiano de Buenos Aires, Buenos Aires, Argentina

**Keywords:** Hereditary renal cell carcinoma, genetic testing, early-onset renal cell carcinoma, VHL, Age criteria in Hereditary renal cell carcinoma

## Abstract

Although age younger than 46 years has been an independent criterion for genetic testing in hereditary renal cell carcinoma (hRCC), there is a lack of evidence in the literature. This study aims to analyze whether a 46-year-old cut-off should be considered an independent genetic testing criterion and to elucidate risk factors predicting a positive genetic test. Observational study from January 2010 to December 2021. All patients under 46 years with a non-metastatic kidney mass and surgical indication were included. We assume patients who relapse in the first 5 years of follow-up could have a positive genetic test. As risk factors for relapse, ergo positive genetic test, we consider those patients who presented multifocal, bilateral, or previous renal tumor. Of 2,232 nephrectomies for kidney cancer, 301 patients met the inclusion criteria. The median follow-up was 60 months (IQR 29-101). The estimated five-year RFS was 94.4% (95% CI 91.3-97.5). Tumor size, previous renal tumor, multifocality, bilaterality, and pT3 or pT4 stage were independent recurrence risk factors. Genetic testing was performed on 24 patients. 10 patients had pathogenic variants in the test, 8 of which recurred during their life. 46-year-old cut-off has shown low performance in genetic testing. Therefore, we recommend that it be considered only if other hRCC risk criteria exist. Multifocality, bilaterality, and previous renal tumor could predict a positive genetic test.

## Introduction

Renal cell carcinoma (RCC) represents 5% of overall tumors, and it is the third most common genitourinary neoplasm behind prostate and bladder cancer (1). Hereditary renal cell carcinoma (hRCC) represents 4-8% of all RCC (2, 3). There are various inherited syndromes, the most common being Von Hippel-Lindau disease (VHL). Other syndromes include Tuberous Sclerosis complex (TSC), Birt-Hogg-Dubé syndrome (BHD), Hereditary leiomyomatosis and RCC (HLRCC), Succinate dehydrogenase-related RCC (SDH), Hereditary papillary RCC (HPRC) among other less common entities. There are multiple genes involved in hRCC (Table 1).

**Table 1:** Genes involved in hRCC, and clinical features.

Syndrome	Gene identified	Renal tumors	Other Finding
Von Hippel- Lindau	*VHL*	Clear cell	Pheochromocytoma, Epididymal tumor, Inner ear tumor, Pancreatic cysts, Retinal hemangioma, CNS hemangioma
Tuberous Sclerosis Complex	*TSC1; TSC2*	AML, Clear cell*	Angiofibromas, Hypopigmented macules, CNS hamartomas, Epilepsy, Cardiac rhabdomyoma, Lymphangioleiomyomatosis
Birt-Hogg-Dubé	*FLCN*	Chromophobe, oncocytoma	Lung cysts, Pneumothorax, Cutaneous fibrofolliculomas
Hereditary Leyomiomatosis and Renal Cell Carcinoma	*FH*	Papillary type II	Cutaneous leiomyoma, Uterine leiomyoma
Hereditary Paraganglioma/ Pheochromocytoma Syndrome	*SDHA/B/C/D*	Both malignant and benign	Paraganglioma, Pheochromocytoma
Hereditary Papillary Renal Cell Carcinoma	*Met*	Papillary type I	None

* younger age, primarily in women.

CNS: Central nervous system; AML: Angiomyolipoma.

There is consensus to perform genetic testing for hRCC when patients are younger than 46 years (early-onset RCC), and/or the presence of multifocal or bilateral masses, and/or patients with familial history of hRCC (4). Furthermore, hRCC can be suspected when extrarenal manifestations are present (e.g., cerebellar hemangioblastomas in VHL, pulmonary cysts in BHD) (4, 5). Genetic testing might include between 15 to 20 relevant genes (6), and it is recommended to perform it as soon as possible, given the personal and familiar implications of these hereditary diseases.

The relevance of the genetic diagnosis is important given that the natural history of these diseases is the recurrence (7–9). Thus, when feasible, the therapeutic approach must be conservative (active surveillance, nephron-sparing surgery, ablation therapies) (9, 10).

Although age younger than 46 years has been a criterion for genetic testing by itself, there is a lack of evidence, and these current recommendations are based on expert opinions, consensus, or low evidence manuscripts (3, 11–13). It is important to perform prospective studies with a 100% rate of genetic testing to find more substantial evidence for this criterion. The economic and psychological costs of genetic testing might not surpass the probable clinical benefits.

This study aims to analyze whether a 46-year-old cut-off should be considered an independent genetic testing criterion, to elucidate risk factors to predict a positive genetic test, and to evaluate the clinical presentation of RCC in patients under 46 years old.

## Material and methods

Observational study of a cohort of patients surveyed from our prospectively collected database from January 2010 to December 2021. All patients under 46 years with a kidney mass and surgical indication were included to answer our hypothesis. Patients who did not undergo surgery or had metastatic disease before surgery were excluded.

We assume that patients who relapse in the first 5 years of follow-up could have a positive genetic test due to the natural history of recurrence in these diseases. As risk factors for relapse, ergo positive genetic test, we consider those patients who presented multifocal, bilateral, or previous renal tumor, besides other variables in the study. Therefore, we divided patients into two groups: patients with a multifocal, bilateral, or personal history of previous renal tumor (Group 1) and patients with a single renal mass (Group 2). The relapse rate in this population under 46 years old will be considered a positive genetic test; therefore, there is a risk of this possibility.

Demographic data, as well as pre, intra, and postoperative variables, were analyzed:

- *Demographic and preoperative data*: Age, gender, comorbidities (diabetes, hypertension, cardiovascular diseases, chronic kidney failure, obesity, immunosuppression, others), risk factors (smoking, dialysis, previous kidney tumor), clinical presentation (incidental or symptomatic), affected side, number of tumors, tumoral size.
- *Intraoperative data*: Type of surgery (partial, radical, bench surgery and autotransplantation, unresectable). Surgical approach (conventional or minimally invasive, either laparoscopic or robot-assisted laparoscopic).
- *Postoperative data*: Postoperative complications (Clavien – Dindo), histological type, TNM classification, follow-up time, recurrence, global death, and cancer-specific death, genetic testing.
  Recurrence was defined as new evidence of radiological disease, either on the ipsilateral or contralateral kidney or both local or distant metastasis. As parameters of oncological results, recurrence-free survival (RFS) time was calculated, defining the former as censored data taken as the time elapsed in months from the time of surgery to the last consultation or recurrence. Similarly, for specific or global cancer mortality, the time from surgery to death resulting from kidney cancer or any cause was considered, respectively.
- *Statistical analysis:* According to distribution, continuous variables are expressed as their mean and standard deviation (SD) or median and interquartile range (IQR). For comparison, the Student or Mann-Whitney test is used. Categorical variables are expressed as their absolute value and percentage (%), compared using the chi-square or Fisher test. Univariate Cox regression evaluates recurrence risk by calculating the hazard ratio (HR) and its 95% confidence interval (95% CI). The multivariate analysis was not conducted because of low event incidence and bias risk. To calculate survival, the Kaplan-Meier method is used with an estimate of survival as long as at least 15% of the initial sample is at risk. To compare survival distributions, a log-rank test is performed. A p-value < 0.05 is considered significant, or an HR whose 95% CI includes 1. The software used was SPSS 22.0, IBM Corp, New York, USA™.

The current protocol was approved by the ethics committee from our institution, “Comité de Etica de Protocolos de Investigación” (CEPI 5404).

## Results

**Figure 1:**
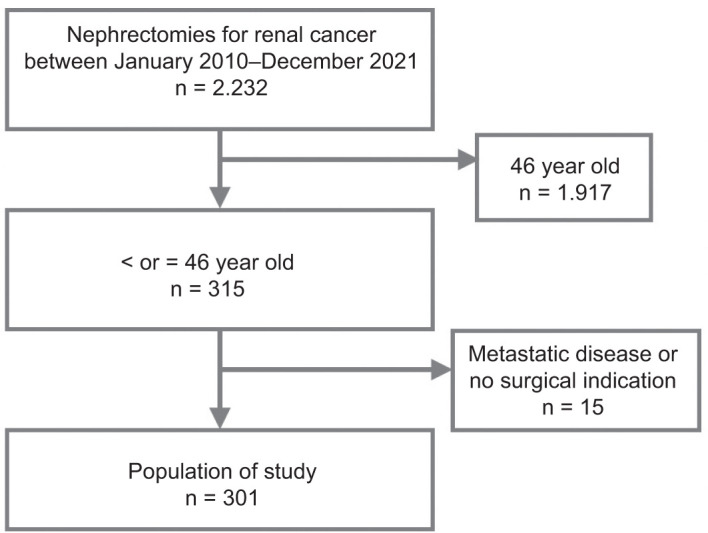
Study population, consort diagram.

Between January 2010 and December 2021, we performed 2,232 nephrectomies for kidney cancer. Three hundred fifteen patients were 46 years old or younger. Fourteen patients had metastatic disease at the diagnosis and were therefore excluded from this study. We did not exclude any other patient because, in all of them, surgery was indicated (n=301) (Figure 1). The median age was 39 (19-46 y.o.). Two hundred twenty patients (73.1%) were male. Seventy-two patients presented with comorbidities. Ninety-nine patients had risk factors, including 12 (3.9%) patients with previous renal tumors. Twenty-four patients had other tumors, the most common papillary tumor of the thyroid (5 patients), with no correlation with hRCC. Four patients who tested positive for VHL had a history of CNS hemangioma. Only four patients had a familial history of renal tumors, and in three, hRCC was diagnosed. Thirty-five patients had a symptomatic tumor. Seven patients (2.3%) had bilateral tumors. Two hundred eighty-one patients had a single renal mass, and 20 had 2 to 9 tumors. The median tumor size was 52 mm. In 124 patients, radical nephrectomy was indicated, partial nephrectomy in 175, 1 patient had bench surgery and auto transplantation, and one had an unresectable tumor. In 242 patients, a minimally-invasive approach was decided (either laparoscopic or robotic-assisted), 57 had open surgery, and conversion was needed in 2 patients. Fifty-two postoperative complications were recorded, and 75% were Clavien 3a or below. Clear cell Renal Cell Carcinoma (ccRCC) was the most common pathological finding (213; 70.8%), followed by chromophobe (19; 6.3%), oncocytoma (15; 5.0%), and others. The TNM classification was: pT1a or b: 210 (79.0%); pT2a or b: 31 (11.7%); pT3 and 4: 25 (9.4%); pN1: 4 (1.3%).

Oncologic outcomes: The median follow-up was 60 months (IQR 29-101). The crude recurrence rate was 10.2% (21 patients). Nine recurred on the homolateral or contralateral kidney, and 15 had systemic recurrence in some patients after kidney recurrence. Thirteen patients started systemic therapy. Eight patients (2.7%) died during the follow-up, six due to kidney cancer, and 2 for other causes. The estimated five-year RFS was 94.4% (95% CI 91.3-97.5).

Recurrence risk factors: Univariate analysis by Cox regression is presented in Table 2. Tumor size, previous renal tumor, multifocality, bilaterality, and pT3 or pT4 stage were independent recurrence risk factors.

**Table 2:** Recurrence risk factors.

	Univariate analysis	
	HR (95%CI)	p
Age (by year)	1.04 (0.96-1.13)	0.281
Male	0.44 (0.13-1.5)	0.190
Comorbidities	0.74 (0.25-2.21)	0.591
Size (by mm)	1.02 (1.01-1.03)	**0.0001**
Previous renal tumor	4.63 (1.36-15.75)	**0.014**
Multifocality	1.46 (1.17-1.82)	**0.001**
Bilaterality	4.99 (1.16-21.5)	**0.031**
pT	—	—
pT1	Ref	Ref
pT2	2.59 (0.69-9.78)	0.159
pT3 or pT4	5.16 (2.03-13.1)	**0.001**

**Figure 2:**
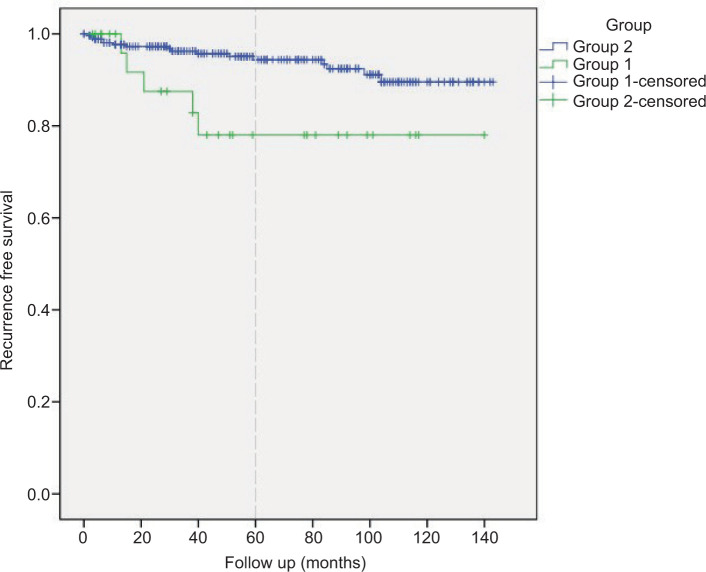
Recurrence-free survival.

The estimated five-year RFS in Group 1 was 78% (95% CI 61-87), while for patients in Group 2, RFS was estimated at 94.4% (95% CI 91.3-97.5-Log rank test p 0.015- Figure 2). Patients in Group 1 have an HR of 3.23 (95% CI 1.18-8.83-p 0.022), predicting recurrence.

Genetic testing was performed in 24 patients: in 14 patients (58.3%), no pathogenic/probably pathogenic variants were identified, 6 (25.0%) had pathogenic variants in VHL, 3 (12.5%) had pathogenic variants in TSC1 or TSC2, and one patient had an ATM gene mutation, with unknown clinical relevance. Of the 14 patients with no variants in the genetic test, only 3 presented bilateral, multifocal, or previous history of renal tumor. Four patients of the 10 with positive genetic tests had a personal history of previous renal tumor, five were suspected of having a congenital disease due to familial history or extrarenal manifestations, and only one patient presented with a single renal mass, but this was the patient with the ATM mutation. Eight patients with pathogenic variants in the genetic test recurred during their life, three during our follow-up, and five were referred to our institution after the diagnosis of relapse. The two patients that did not relapse were one with TSC and one with the ATM mutation. Analyzing by groups, Group 1 included 11 patients (8 with pathogenic and three without pathogenic variants), and Group 2 included 13 patients (2 with pathogenic variants and 11 without pathogenic variants). The Positive Predictive Value was 73% for Group 1 and 15% for Group 2, whereas the Negative Predictive Value was 85% for both groups.

## Discussion

This study represents a large cohort in a single institution in Argentina with a multidisciplinary VHL unit, recognized as a Clinical Care Center of the VHL Alliance (www.vhl.org). In addition, our Urology Department is a national referral center for kidney cancer, with a high surgical volume. Since genetic testing criteria for patients under 46 years old has been discussed since around 2013, and our database dates from 2010, we decided to consider patients with probable positive genetic tests to those patients who relapsed during the first five years of follow-up.

Age criteria for genetic testing are based on a publication by Schuh et al. (3), who evaluated the age distribution of 106,224 patients in the SEER-7 database. The 10th percentile of the overall RCC age was 46 years old. Analyzing 608 patients of the NCI protocol database with hRCC, they identified that a cut-off point of 46 years old would improve genetic test performance. Although the large number of patients presented in this study, only 0.57% of the population analyzed had hRCC. Our findings suggest that performing a genetic test on patients with a single renal mass who are 46 years old or younger as single criteria has low performance, given that we estimated 94.4% of patients free of recurrence at five years. Thus, we assume a probable negative genetic test.

It may be arguable that recurrence equals a positive genetic test, given that we have few patients tested, but in our database, all the patients with genetically confirmed hRCC relapsed during the first five years of follow-up, and therefore we believe that our interpretation is valid.

Maher (5) stated that testing individuals with low risk for a mutation can lead to identifying genetic mutations of uncertain clinical significance. In our study, we diagnosed a patient with ATM gene mutation, but the clinical relevance of this is not clear. Lui et al. (4) suggested that when the risk is very low, they do not advocate for testing, especially when the patient has concerns regarding its potential risks. Genetic testing might have a significant emotional impact. Although it has not been studied in hRCC, there is plenty of evidence of the psychological reactions that might occur in patients facing a possible hereditary disease. Ponzone et al. (14) analyzed the impact on patients offered genetic testing for hereditary breast cancer. They found that 80% of women with a positive test presented anxiety and depression, and 1% considered suicide. Besides, 42% of patients with a negative result were still worried after the test, and 25% felt guilty concerning affected members. Kessler et al. (15) studied relatives of patients with Huntington’s disease and their response to genetic counseling and testing. Eighty-three percent of the subjects said they would limit their reproduction if the test were positive, and 11% said they would consider suicide. Thirty-seven percent of the subjects reported that an immediate family member had attempted suicide after their relatives’ diagnosis, and 34% were hospitalized for psychiatric reasons. This psychological impact must be taken into account when offering a genetic assessment. In our institution, we would have tested 94.4% of the patients, with its consequent psychological risk, without an evident clinical benefit.

Kokorovic et al. (16) investigated clinical predictors for a positive test result in hRCC. They assessed 74 patients who were tested and found that dermatological findings and two or more high-risk criteria were the only predictors for a positive test result. It must be highlighted that from the five patients with a positive impact, only one was younger than 46 and presented three genetic testing criteria. They stated that as outlined by current guidelines criteria, most patients with high-risk features will not give a positive genetic test. In our 46 years old or younger population, we find that previous renal tumor, multifocality, or bilaterality significantly increases the chance to recur, making these patients candidates to perform a genetic test. These findings reinforce our belief that age should not be an independent criterion.

Ideally, we should design a study with a 100% rate of genetic testing to evaluate the test performance accurately. Our study only tested 24 patients (8.0%), an issue common currency in most papers in the literature. Kushnir et al. (17) applied the Canadian hereditary RCC risk criteria to their prospective database. From the 8,388 patients enrolled, 2,827 (35%) met at least one risk criterion. Fifty-six of the at-risk patients were genetically tested (2.0%). They found that 35% had a germline mutation, but they did not mention which mutations were or whether they were clinically relevant. Although we may not present overwhelming evidence, our genetic testing rate could be the highest published, only selecting patients by age.

We must mention some limitations of our study. Since our database started in 2010, genetic testing was only available for a few patients. Another issue is that genetic testing is only sometimes accepted by insurance companies, and some patients cannot afford the costs of the test. Furthermore, as a referral center with several patients that return to their provinces after surgery, the loss of follow-up in some patients may make it difficult to collect the data. Our VHL unit is working on these aspects to be able to present prospective studies with genetic testing in the future.

## Conclusion

Age by itself has shown low performance in genetic testing. Therefore we recommend that the 46-year-old cut-off be considered only if other hRCC risk criteria exist. Multifocality, bilaterality, and previous renal tumor could predict a positive genetic test. A small percentage of patients present early-onset RCC, and most showed a single renal mass at the diagnosis.
